# Advancing the application of systems thinking in health

**DOI:** 10.1186/1478-4505-12-50

**Published:** 2014-08-26

**Authors:** Taghreed Adam

**Affiliations:** Alliance for Health Policy and Systems Research, World Health Organization, 1211 Geneva 27, Switzerland

## Editorial

### Highlighting the growing significance of systems thinking in health: introducing a new global series

**Figure 1:**
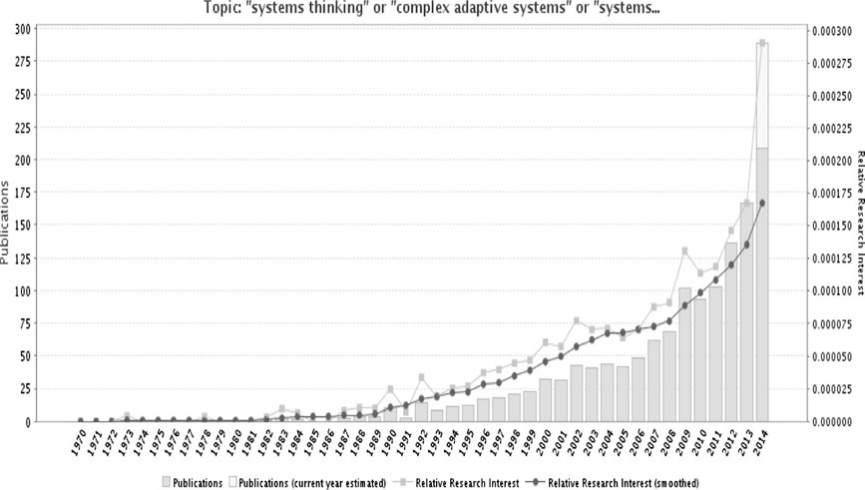
**Trends in the use of the terms “systems thinking”, “complex adaptive systems”, or “systems science” in the Medline database over the past 40 years.** Source: GoPubMed, which reports the frequency that terms appear in MEDLINE indexes for publications, which include titles, abstracts, journal names and corresponding author’s affiliation. Number of publications mentioning these search terms was 1386 as of 14 August 2014.

**Figure 2:**
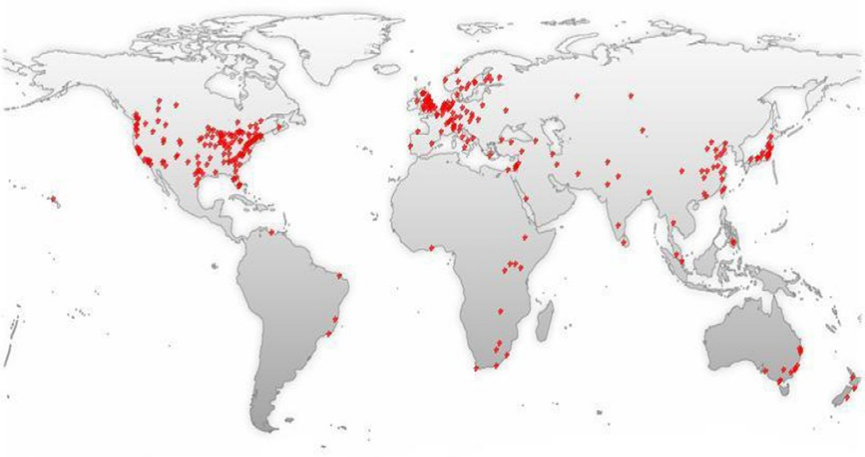
**World map of the 1,386 MEDLINE records mentioning the terms “systems thinking”, “complex adaptive systems”, or “systems science”.** Source: GoPubMed, which reports the frequency that terms appear in MEDLINE indexes for publications, which include titles, abstracts, journal names and corresponding author’s affiliation. This data was obtained on 14 August 2014.

The past two decades have witnessed an increased recognition among global health stakeholders of the importance of systematically considering the complex adaptive nature of health systems to better anticipate some of the unexpected and counterintuitive consequences of implementing current and new policies. This is evidenced by the increased interest in topics such as systems thinking, complex adaptive systems, and systems science in the published health literature over the past 20 years (Figure 1). However, the majority of these publications are from high-income countries, while the need for applying these concepts is at least as great in low- and middle-income countries (LMICs) (Figure 2). Most of these studies discuss the concepts or make the case for the utility of systems thinking for health systems strengthening; there is still a dearth in practical guidance on how systems thinking concepts, approaches, and tools can be applied in health systems research and practice to reach sustainable solutions [1, 2].

Systems thinking is, foremost, a mindset that views systems and their sub-components as intimately interrelated and connected to each other, believing that mastering our understanding of how things work lies in interpreting interrelationships and interactions within and between systems [1, 3, 4]. It is a perspective that deliberately goes beyond events, to look for patterns of behavior and the underlying systemic interrelationships which are responsible for these patterns and their associated events [5]. It embraces the understanding of open systems as complex adaptive systems that are constantly changing, resistant to change, counter-intuitive, non-linear, and where the whole is greater than the sum of the parts [3].

The Alliance for Health Policy and Systems Research (hereafter called the Alliance) has been one of the avid advocates for moving this kind of thinking forward, dedicating a number of activities and resources to promote this field among health practitioners and researchers. First, through its flagship publication on “*Systems Thinking for Health Systems Strengthening*” in 2009 [5], followed by a Journal Supplement in *Health Policy and Planning*, in 2012, it has sought to generate better understanding of current practices in applying systems thinking for health systems in LMICs [1].

The 2012 supplement demonstrated the dearth of applications that explicitly took into account the complexity and dynamics resulting from intervening in health systems, including evaluations of interventions with system-wide effects [2]. In addition, the very few applications that existed at the time of developing that supplement were predominately from high income countries [1]. These observations revealed the need for concerted efforts to advance the application of systems thinking in health, particularly in LMICs.

In March 2013, the Alliance, in collaboration with Canada’s International Development Research Centre, launched a Call for papers inviting teams of researchers and health practitioners, with particular focus on lead authorship from LMICs, to develop and share applications of systems thinking methods and approaches, culminating in this Series. This whole program of work, which spanned over two and a half years, provided a great opportunity for strengthening programs, policies, and methods in LMICs to enable researchers and decision makers to think through how systems thinking approaches can be applied to their current health systems questions with practical results.

It is worth noting that, while this collection of articles offers innovative and diverse range of applications of systems thinking approaches, methods, and tools, as the Commentary by Peters illustrates [6], these applications by no means capture the entire range of relevant tools and approaches that can be applied.

### The applicability of a wide range of tools and approaches

This Series illustrates how research approaches that are commonly used in various disciplines, such as realist evaluation, sense-making (as a mental model), or program evaluation theories, can be applied within a systems thinking approach to address complex health systems questions. It does so by showing that the types of questions asked are the most important element that shape the orientation of the analysis, not the tool itself.

For example, in the paper by Prashanth et al. [7], systems thinking and complex adaptive systems approaches added depth to the realist evaluation by digging deeper into the drivers of, and the context in which the differences in responses of health workers in the two sub-districts were observed and what triggered them. They could show that settings with committed staff and positive intentions to make changes demonstrated more positive outcomes and an ability to use existing opportunities to solve problems and improve performance. Further, that commitment alone was neither crucial nor sufficient as demonstrated by findings from another setting with committed staff but different outcomes. Finally, that in settings with a lack of commitment from staff, strong leadership became more pronounced in driving the change into better outcomes [7].

### Systems thinking and mixed methods

As discussed by Peters and demonstrated by several of the Series papers, both qualitative and quantitative methods contribute in their own way to our understanding of complexity [6, 8]. As some of the early systems thinking literature originated from quantitative disciplines such as physics and biology, it may give the impression that relevant systems thinking approaches are predominantly quantitative. Perhaps one of the main contributions of this Series is demonstrating how qualitative methods commonly used in fields such as social science or anthropology add equally important value and depth to analyses of complex health systems questions and phenomena [8–12]. For example, they are often used to provide a profound initial understanding of the problem that can then be complemented by quantitative approaches that incorporate the learning into a more realistic and sophisticated quantitative analysis [6].

### Exploiting the potential of visual interpretations of complex phenomena

During the past decade there has been a revolution of infographics due to the increased recognition of the power of graphics to aid data interpretation and decision making. In this Series, several papers illustrate how a range of graphic tools can help convey complex interpretations and findings in a meaningful visual form, namely causal loop diagrams [8–12], stakeholder network analysis, and sociographs [13, 14].

For example, causal loop diagrams used by Rwashana et al. to understand the causes of neonatal mortality in Uganda not only helped analyze and make sense of the different sources of data in a dynamic and iterative way, they were also used to present these complex findings in one main diagram that summarized the relationships, dynamics, and associated factors all in one graph [8].

Another example of visual interpretations is presented in Malik et al., where they used sociographs to interpret the pattern of advice-seeking behavior among primary health care physicians and the potential explanations for their choices [13].

### Content of the series

The Series covers a range of systems thinking methods, tools, and approaches, including system dynamics modeling [15], causal loop diagrams [8–12], and social network analysis [13, 14]. In addition, several papers couched their analysis in a complex adaptive systems framework [9, 10, 16], or adapted established methods, such as realist evaluation [7, 12] and policy analysis [16, 17], to untangle the underlying complexity of their research questions. The main approaches, research questions. and findings of the Series papers are discussed in turn below.

The paper by Bishai et al. uses a system dynamics simulation model to illustrate trade-offs and unintended consequences in allocative funding decisions to curative versus preventive care [15]. The model provides a quantitative application of complex adaptive systems methodologies to a health systems and policy question, something that traditional cost-effectiveness analysis techniques fail to illustrate. In this paper, the authors demonstrate how the growth of curative care services can crowd both fiscal space and policy space for delivering population-level prevention services, which would require extensive and long-term interventions to overcome the fiscal and population health consequences [15].

The paper by Prashanth et al. is one of three papers exploring capacity strengthening initiatives targeting health workers and managers [7]. They use realist evaluation to explore how a capacity building intervention for district health managers implemented in two different places evolved over time, taking into account the context and the mechanism of change. The paper highlights the importance of the people involved and the choices they make in the evolution of outcomes, and how individual and organizational attributes and the interaction between them contribute to any particular outcome [7].

Kwamie et al. is another paper looking at capacity strengthening of middle-range managers [12]. The authors also used realist evaluation, supplemented by visually interpreting their findings using a causal loop diagram, to examine how and why a Leadership Development Programme works when it is introduced into a district health system and whether or not it supports systems thinking in district teams, using Ghana as a case study. They conclude that the leadership program on its own did not lead to the development of a systems thinking approach in management and decision-making in the district and argue that the complexity of organizational contexts and history are important influencing factors for the sustainability or scaling up of such programs, as much as the complexity of the intervention itself [12].

Gilson et al. stimulate wider thinking about the forms and practices of health leadership [17]. They use the concepts of sense-making and discretionary power drawn from the theories of complex adaptive systems and policy implementation to highlight how important it is that health system actors are able to make sense of the intentions of policies to be able to incorporate them into their everyday routines and practices. The study reveals how the collective staff understanding of their working environment, and how changes occur within it, may act as a barrier to centrally-led initiatives to strengthen capacities [17].

Next, in an application of social network analysis, the study by Malik et al. describes the formal and informal ways in which primary care physicians in Pakistan access information [13]. By employing a range of research methods, the paper examines the reasons for the disparity between organizational structures for supervisory and reporting relationships and the actual behavior of primary care physicians when seeking information. They argue for the importance and value of exploring the supervisory and technical support arrangements from the view point of the users [13].

In the paper by Paina et al. [9], the authors investigate how, in a context of no official government policy on dual practice, this practice is currently being regulated in Uganda through a system of “unwritten” expectations and local management practices that have not been elsewhere documented. Through a series of causal loop diagrams and historical and primary accounts, the authors depict the resulting behavioral patterns and complex systems characteristics such as policy resistance in the form of protests by government providers and coping approaches by providers and their managers to maintain public sector’s service delivery and performance [9].

Rwashana et al. offer another application of causal loop diagrams in exploring the complexity which characterize neonatal health and its interplay with health systems factors, using Uganda as a case study [8]. The analysis revealed multiple feedback loops, such as trust, that household place on the health system, awareness of the benefits of antenatal and postnatal care, myths, frustration of health workers and its impact on all aspects of their performance, among others. The authors also discuss high leverage points that may be considered by policy makers to improve neonatal health such as gender considerations related to girls education and empowerment [8].

Next, in their analysis of the causes of reductions in coverage of vaccination in Kerala, Varghese et al. demonstrate how important it is that the evidence used to design and evaluate public health programs goes beyond epidemiological and economic analysis [10]. The paper shows how key factors that contributed to the unexpected decline in vaccination coverage were revealed such as how the opposition by the government medical doctors association and alternative medicines proponents, compounded by strong media influence, have evolved overtime and created a big unexpected influence on people’s decision to vaccinate [10].

The following two papers explore experiences with two financing schemes in Ghana and China. In their analysis of the Ghana National Health Insurance Scheme, Agyepong et al. suggest that relatively less attention seems to be paid to service access and service responsiveness when evaluating an insurance scheme [11]; something that people thinking about enrolling consider as much as the financial risk protection potential. They highlight why a comprehensive systems thinking approach is essential when conceptualization and designing a health insurance scheme to avoid the emergence of counterintuitive and undesired effects [11]. Zhang et al. offer another perspective of a financing intervention, by exploring the evolution of rural finance schemes in China [16]. The paper discusses the nature of health systems resilience facing the implementation problems associated with the policy and argues that initial trajectories have been a big determinant of how policy-makers adapted in the various contexts [16].

Blanchet et al. examine sustainability in internationally-initiated programs in their comparison of sustainability-oriented processes in two rehabilitation centres in Nepal and Somaliland [14]. The paper shows how differences in the governance and network structure of the rehabilitation centres, revealed through a stakeholder network analysis, have influenced the process and commitment to sustainability. The analysis helped in the understanding of change in the nature of relationships between actors and their capacity to work together over time and what factors are conducive to providing the right incentives to work as an interrelated network rather than as individual actors [14].

In second paper addressing sustainability issues, Sarriot et al. examine how an international NGO worked with two Bangladeshi municipal health departments to intentionally advance sustainability in their support for maternal and child health preventive services [18]. The paper explores how systems thinking was used to generate a process of change within municipal health systems, affecting technical, social, political, and organizational sub-systems. The authors document how a sustainability framework method was used to work with stakeholders in an explicit process to guide their decisions and choices during and after the life of a project. They illustrate how this process offered useful tools to engage stakeholders, give shared meaning to information about activities and achievements, facilitate decision making, and mitigate the risk of unintended project effects in order to achieve a measure of sustainability in a complex setting [18].

Last but not least, the commentary by Peters discusses which of the large body of theories, tools, and methods associated with systems thinking are more useful to understanding the behaviour and complexity of health systems [6]. It also discusses the “jungle” of terminology surrounding this field and how and why some terms have emerged and been used differently in different disciplines. It then provides a helpful overview of a wide range of systems thinking theories, methods, and tools that are relevant to understanding and exploring health systems questions [6].

### Looking forward

With the selection of papers in this Series, our aim was to give meaning to abstract concepts and theories through actual applications and experiences of how systems thinking tools and concepts can be used to understand and strengthen health systems, particularly in LMICs. We hope that by providing a variety of experiences, examples, and ideas that are relevant to other complex interventions and contexts, this collection will stimulate wider applications and innovations of these and other approaches relevant to this field.
